# Controlled synthesis and luminescence properties of core-shell-shell structured SiO_2_@AIPA-S-Si-Eu@SiO_2_ and SiO_2_@AIPA-S-Si-Eu-phen@SiO_2_ nanocomposites

**DOI:** 10.1038/s41598-020-60538-w

**Published:** 2020-02-26

**Authors:** Yan Qiao, Wenxian Li, Jinrong Bao, Yushan Zheng, Lina Feng, Yangyang Ma, Kuisuo Yang, Anping Wu, He Bai, Yunjiang Yang

**Affiliations:** 1 Inner Mongolia Key Laboratory of Chemistry and Physics of Rare Earth Materials, School of Chemistry and Chemical Engineering, Inner Mongolia University, Hohhot, 010021 China; 2 Inner Mongolia Autonomous Region Food Inspection Test center, Hohhot, 010021 China

**Keywords:** Chemistry, Materials science

## Abstract

Two novel core-shell structured SiO_2_@AIPA-S-Si-Eu and SiO_2_@AIPA-S-Si-Eu-phen nanocomposites have been synthesized by a bifunctional organic ligands ((HOOC)_2_C_6_H_3_NHCONH(CH_2_)_3_Si(OCH_2_CH_3_)_3_) (defined as AIPA-S-Si) connected with Eu^3+^ ions and silica via covalent bond. And the corresponding core-shell-shell structured SiO_2_@AIPA-S-Si-Eu@SiO_2_ and SiO_2_@AIPA-S-Si-Eu-phen@SiO_2_ nanocomposites with enhanced luminescence have been synthesized by tetraethyl orthosilicate (TEOS) hydrolysis co-deposition method. The composition and micromorphology of the nanocomposites were characterized by means of Fourier-transform infrared spectroscopy (FT-IR), thermal gravimetric analysis (TG), X-ray diffraction (XRD), scanning electron microscopy (SEM), transmission electron microscopy (TEM), energy-dispersive X-ray spectrometry (EDX) and X-ray photoelectron spectroscopy (XPS). The as-synthesized core-shell and core-shell-shell structured nanocomposites have excellent luminescence intensity and long lifetime. The nanocomposites show bright red light under ultraviolet lamp. However, the core-shell-shell structured nanocomposites have stronger luminescence intensity than the corresponding core-shell structured nanocomposites. Meanwhile, the core-shell-shell structured nanocomposites still exhibit good luminescence stability in aqueous solution. In addition, a large number of Si-OH on the surface of the core-shell-shell structured nanocomposites can be attached to many biomacromolecules. Therefore, they have potential applications in the fields of biology and luminescence.

## Introduction

In recent years, core-shell structured nanocomposites are gaining increasing attention due to their unique structures and properties. Core-shell structured nanocomposites not only have core properties but also shell properties, or new properties depend on the interaction between the core and the shell. This unique property also makes it a very important emerging nanomaterial that is widely used in various fields. For example, bio-nanotechnology, optics, electroluminescence devices, bio-imaging energy storage materials, etc.^1–8^.

Silica is commonly used as core and shell material in core-shell structured nanocomposites due to its high mechanical stability, low cost, low cytotoxicity, ease of preparation, and bioconpatibility^9–12^. So far, many research groups have studied core-shell structured nanocomposites using silica as core and shell, which have potential applications in different fields. Yan’s group had reported the SiO_2_@lanthanide complex@MOF composites with excellent properties for metal ion sensing^13^. Li’s group had reported the SiO_2_@Tb/GMP as fluorescence sensor probe^14^. Tobias’s group had reported that SiO_2_@Pt@SiO_2_ is used as a new type of optical component^15^. In previous studies, our group reported that the core-shell structured nanocomposite SiO_2_@MABA-Si-Tb was synthesized by using MABA-Si as a ligand. It is found that SiO_2_ can improve the luminescence intensity and stability of rare earth complexes, which has potential application prospects in the field of luminescence^16^.

Over the years, though the core-shell structured nanocomposite with silica coating inorganic materials were successive studied^17–19^, but their weaker luminescent properties limit their application in many fields. However, the rare earth organic complexes have excellent luminescence properties, which the energy of the organic ligands was efficiently transferred into the rare earth ions by “antenna effect”^20^. Besides, rare earth organic complexes have strong and narrow emission bands and are widely used in many optical fields. Unfortunately, rare earth organic complex themselves are still restricted from being utilized in practical applications due to their poor thermal and mechanical stability^13^. If the rare earth organic complex were immobilized on the surface of the silica, the silica core can reduce the skeleton vibration of the ligand, thereby effectively improving the stability and luminescence intensity of the complex^21–23^. The nanocomposites with silica as the core and rare earth complexes as the shell have good luminous properties, which make them more widely used. However, in the core-shell structured nanocomposites, the rare earth organic complex of the outer shell layer being exposed to the environment, which tends to quench the luminescence. In order to overcome this problem, the core-shell structured nanocomposite is coated with an amorphous silica to form a core-shell-shell structured nanocomposite, which can improve the luminescent properties of nanocomposites. The core-shell-shell structured nanocomposites designed in this way has excellent luminescence properties and good stability. In addition, the SiO_2_ shell coated on the surface of the core-shell structured nanocomposite is easily condensed with the -OH group on other molecules to form a Si-O-Si network, and various functional groups are easily introduced into the silica shell, which is widely used in biomedicine^24–26^.

Up to now, there are two main methods for preparing core-shell structured nanocomposites: direct precipitation method and silane coupling agent method. Direct precipitation method is to deposit functional shell materials directly on the surface of SiO_2_ core. The method is simple to operate, but the prepared nanocomposites are unstable and prone to collapse. The silane coupling method is to join the SiO_2_ core and the shell by a covalent bond. What’ more, the shell layer thickness of nanocomposites is easy to control and structurally stable via this method. The realization of this method is challenging. It was have a silane coupling agent called a “molecular bridge” to bond rare earth ions and SiO_2_. Accordingly, it is important to choose a suitable silane coupling agent.

In this work, firstly, we synthesized the bifunctional organic ligands ((HOOC)_2_C_6_H_3_NHCONH(CH_2_)_3_Si(OCH_2_CH_3_)_3_, it is obtained by single-substitution of hydrogen atom on the amino group of AIPA by TEPIC. Defined as AIPA-S-Si) using 5-amino-phthalic acid (AIPA) and the silane coupling agent 3-(triethoxysilyl)-propyl isocyanate (TEPIC). The as-prepared ligand (AIPA-S-Si) has two carboxyl groups, which are easier to coordinate with rare earth Eu^3+^ ions so that the synthesized nanocomposites have excellent luminescence properties. Then four kinds of nanocomposites SiO_2_@AIPA-S-Si-Eu, SiO_2_@AIPA-S-Si-Eu-phen, SiO_2_@AIPA-S-Si-Eu@SiO_2_ and SiO_2_@AIPA-S-Si-Eu-phen@SiO_2_ were successfully prepared by the bifunctional organic ligands (AIPA-S-Si). The luminescence spectra indicated that the as-synthesized nanocomposites have strong luminescence properties. Because SiO_2_ shell can protect core-shell structured nanocomposites from the influence of external environment, the luminous efficiency of core-shell-shell structured nanocomposites are higher than that of the corresponding core-shell structured nanocomposites. The as-synthesized core-shell and core-shell-shell structured nanocomposites greatly reduce the amount of rare earth and the production cost is reduced in practical application. More importantly, many Si-OH groups on the surface of core-shell-shell structured nanocomposites can be easily connected with biomolecules, which can be widely studied in the field of biology.

## Results and Discussion

### Formation mechanism of core-shell and core-shell-shell structured nanocomposites

**Scheme 1:**
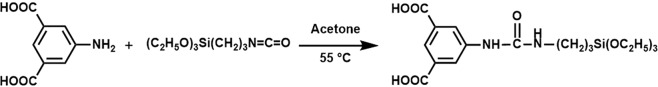
The synthesis mechanism of ligand AIPA-S-Si.

**Scheme 2:**
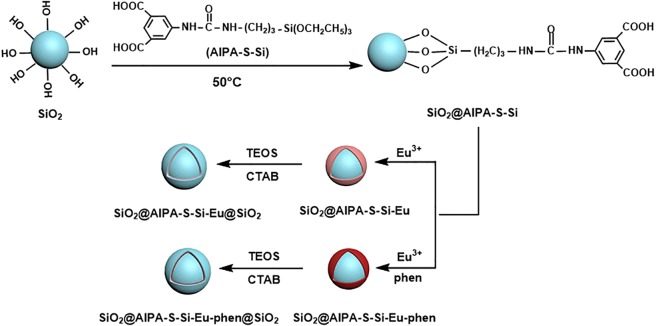
The synthesis mechanism of core-shell and core-shell-shell structured nanocomposites.

The synthesis mechanism of the ligand AIPA-S-Si was shown in Scheme 1. Specifically, the bifunctional ligand AIPA-S-Si was obtained by linking an amino group of AIPA having carboxyl group and isocyanate group of TEPIC having ethoxy group. From the structure of the ligand AIPA-S-Si, the carboxyl group on one side of the ligand is coordinated with Eu^3+^, and the ethoxy group on the other side is connected by hydrolysis and condensation with the silanol group on the surface of the SiO_2_ microspheres. The synthesis mechanism of core-shell and core-shell-shell structured nanocomposites were shown in Scheme 2. In this work, SiO_2_ was a core, AIPA-S-Si was the first ligand, and phen was the second ligand. The SiO_2_ was modified by hydrolysis and condensation of silanol groups on the surface of the silica microspheres and alkoxy groups on the first ligand AIPA-S-Si. A mount of carboxylic group exposed on the surface of the modified SiO_2_. Then, the Eu^3+^ ions coordinated with the carboxylic oxygen atoms of ligand (AIPA-S-Si) on the surface of the modified SiO_2_ and nitrogen atoms of second ligand phen. The core-shell structured nanocomposites were formed by “molecular bridge”. Finally, the core-shell-shell structured nanocomposites were prepared by TEOS hydrolyzed on the surface of the core-shell structured nanocomposites under the action of CTAB.

### SEM and TEM analysis of SiO_2_ and SiO_2_@AIPA-S-Si

**Figure 1:**
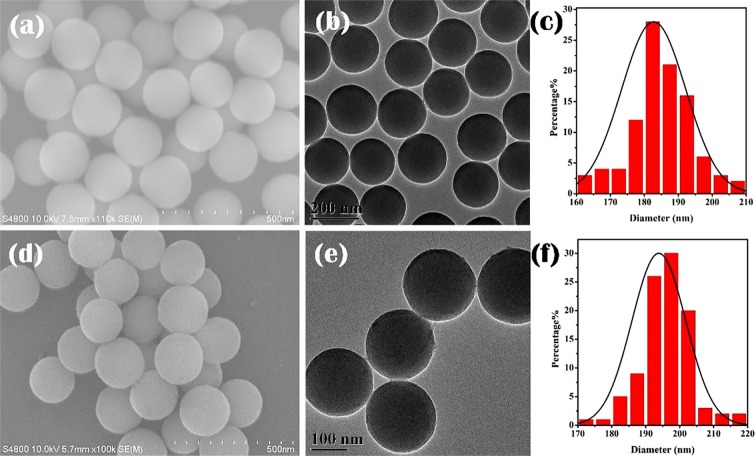
SEM and TEM images of SiO_2_ (**a,b**) and SiO_2_@AIPA-S-Si (**d,e**). Size distribution of SiO_2_ (**c**) and SiO_2_@AIPA-S-Si (**f**).

The morphology and structure of all samples were further investigated by scanning and transmission electron microscopy. Figure 1(a,b) shows the SEM and TEM of SiO_2_ microspheres. It can be observed that SiO_2_ has smooth surface and good dispersibility with an average diameter of about 180 nm. SEM and TEM of SiO_2_@AIPA-S-Si in Fig. 1(d,e) shows that the surface of the SiO_2_ microspheres were slightly rough. Nevertheless, it was worth noting that the size distribution of Fig. 1(c,f) shows that there was a significant difference in diameter between SiO_2_ and SiO_2_@AIPA-S-Si, and the diameter of SiO_2_@AIPA-S-Si increases by about 10 nm compared with SiO_2_ microspheres. The results showed that the organic ligand was successfully grafted onto the surface of the SiO_2_ microspheres.

### TEM analysis of core-shell and core-shell-shell structured nanocomposites

**Figure 2:**
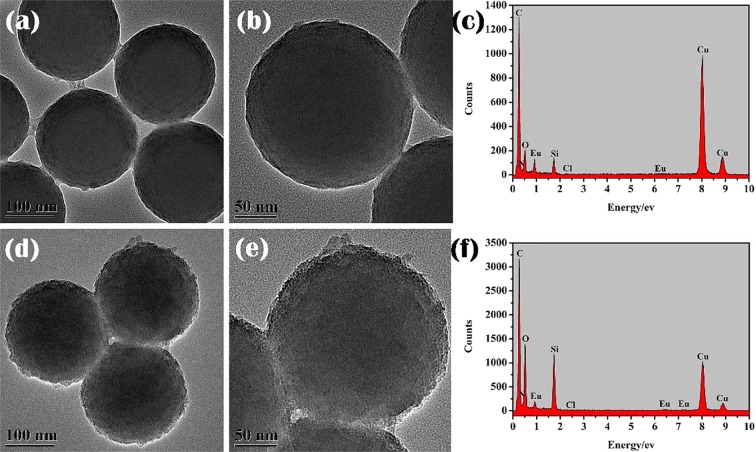
TEM images of SiO_2_@AIPA-S-Si-Eu (**a,b**) and SiO_2_@AIPA-S-Si-Eu@SiO_2_ (**d,e**). EDX images of SiO_2_@AIPA-S-Si-Eu (**c**) and SiO_2_@AIPA-S-Si-Eu@SiO_2_ (**f**).

**Figure 3:**
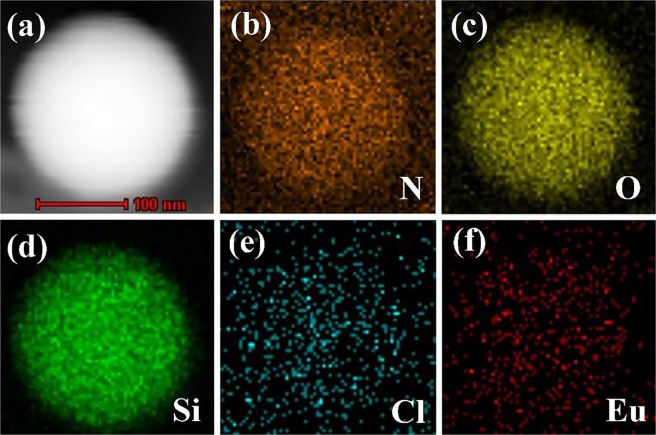
(**a**) HRTEM image of SiO_2_@AIPA-S-Si-Eu@SiO_2_ nanocomposite and corresponding elemental mapping images represent the (**b**) nitrogen mapping, (**c**) theoxygen mapping, (**d**) silicon mapping, (**e**) chlorinum mapping, and (**f)** europium mapping.

The TEM images of SiO_2_@AIPA-S-Si-Eu and SiO_2_@AIPA-S-Si-Eu@SiO_2_ nanocomposites were presented in Fig. 2(a,b,d,e). Accordingly, the TEM images of SiO_2_@AIPA-S-Si-Eu-phen and SiO_2_@AIPA-S-Si-Eu-phen@SiO_2_ nanocomposites were shown in Fig. S1(a,b,d,e). It can be observed from Fig. 2(a,b) and Fig. S1(a,b) that the SiO_2_@AIPA-S-Si-Eu and SiO_2_@AIPA-S-Si-Eu-phen nanocomposites have a rougher surface than SiO_2_@AIPA-S-Si, but still have good dispersibility. Furthermore, the surface roughness of core-shell-shell structured nanocomposites are very different from that of the corresponding core-shell structured nanocomposites. It can be seen from Figs. 2 and S1(d,e) that the gray layer was attached to the surface of the core-shell structured nanocomposites. Specifically, the amorphous silica layer approximately of 8–10 nm thick was successfully coated onto the SiO_2_@AIPA-S-Si-Eu and SiO_2_@AIPA-S-Si-Eu-phen, respectively. In addition, the EDX analysis that was performed on the core-shell and core-shell-shell structured nanocomposites suggested that the existence of N, O, Si, Cl and Eu peaks in the spectra (Figs. 2 and S1(c,f)). The strong peaks of Cu and C that can be observed from the spectra are due to the carbon coated copper grid. It can be found that the contents of Eu in SiO_2_@AIPA-S-Si-Eu, SiO_2_@AIPA-S-Si-Eu@SiO_2_, SiO_2_@AIPA-S-Si-Eu-phen and SiO_2_@AIPA-S-Si-Eu-phen@SiO_2_ nanocomposites were 3.47%, 0.17%, 4.09% and 0.22%, respectively. The content of Eu in the core-shell-shell structured nanocomposite is lower than that of the corresponding core-shell structured nanocomposite, which further proves that the amorphous silica is successfully coated on the surface of the core-shell structured nanocomposite. In addition, TEM mapping measurements of SiO_2_@AIPA-S-Si-Eu@SiO_2_ (Fig. 3) and SiO_2_@AIPA-S-Si-Eu-phen@SiO_2_ (Fig. S2) nanocomposites were performed, which exhibited uniform distributions of N, O, Si, Cl, and Eu components throughout the nanocomposites. The above data further demonstrated the successful synthesis of core-shell and core-shell-shell structured nanocomposites.

### The FT-IR spectra analysis of AIPA-S-Si

The FT-IR spectra further confirmed the synthesis of ligand AIPA-S-Si. Figure S3 shows the FT-IR spectra of the AIPA (a) and AIPA-S-Si (b). For the FT-IR spectra of AIPA (a), bands located at 3425 cm^−1^ and 1695 cm^−1^ were assigned to the stretching vibration of hydroxyl (ν_OH_) and carbonyl groups (ν_C=O_), respectively. The emergence of band located at 1629 cm^−1^ belongs to the stretching vibration of NH (ν_N-H_). In the FT-IR spectrum of AIPA-S-Si (b), the appearance of the new bands located at around 1573 cm^−1^ and 1635 cm^−1^ due to the absorption of amide groups (–CONH–). Besides, the presence of stretching vibrations of methyl (–CH_3_) and methylene groups (–CH_2_) were located at 2977 cm^−1^, 2931 cm^−1^ and 2885 cm^−1^ from TEPIC. In addition, the stretching vibration (ν _Si-O_) located at 1103 cm^−1^ and the stretching vibration (ν _Si-C_) located at 956 cm^−1^ suggested that TEPIC has been successfully grafted onto AIPA^27–32^. The above data indicates that the ligand AIPA-S-Si has been successfully synthesized.

### The FT-IR spectra analysis of SiO_2_@AIPA-S-Si-Eu and SiO_2_@AIPA-S-Si-Eu@SiO_2_

The FT-IR spectra was conducted to confirm the presence of chemical groups in the nanocomposites. At the same time, the synthesis of core-shell and core-shell-shell structured nanocomposites were further confirmed. The FT-IR spectra of SiO_2_, SiO_2_@AIPA-S-Si, SiO_2_@AIPA-S-Si-Eu and SiO_2_@AIPA-S-Si-Eu@SiO_2_ were shown in Fig. S4. In the spectrum of SiO_2_ (Fig. S4a), the characteristic absorption band of Si-O-Si located at 1095 cm^−1^ (ν _Si-O-Si_), 470 cm^−1^ (δ_Si-O-Si_) and Si-OH was identified at 956 cm^−1^ ^33,34^. The characteristic absorption band of the SiO_2_@AIPA-S-Si (Fig. S4b) was located at 1635 cm^−1^ (ν _C=O_) and 1566 cm^−1^ (δ _NH_), which was attributed to the stretching vibration of amide groups (–CONH–). The characteristic bands located at 1697 cm^−1^ belonged to stretching vibration of –C=O– (COOH). This further indicates that the ligand AIPA-S-Si was successfully coated on the surface of the SiO_2_ microspheres. In the FT-IR spectrum of SiO_2_@AIPA-S-Si-Eu (Fig. S4c), the characteristic absorption bands of –CONH– were located at 1627 cm^−1^ and 1558 cm^−1^, respectively. Obviously, compared with SiO_2_@AIPA-S-Si (Fig. S4b), the absorption band of amide groups was red shifted by 8 cm^−1^, indicating that the first ligand AIPA-S-Si coordinated with the Eu^3+^ ions by carboxyl oxygen atom. Fig. S4d shows the FT-IR spectrum of SiO_2_@AIPA-S-Si-Eu@SiO_2_. After coating a layer of amorphous SiO_2_ on the core-shell structured nanocomposite, the characteristic absorption band of SiO_2_ appeared at 1095 cm^−1^ (ν_Si-O-Si_) and 952 cm^−1^ (ν_Si-OH_), and the absorption bands of –CONH– and –C=O– (COOH) were obviously weakened. This further confirmed the synthesis of the SiO_2_@AIPA-S-Si-Eu@SiO_2_ nanocomposite.

### The FT-IR spectra analysis of SiO_2_@AIPA-S-Si-Eu-phen and SiO_2_@AIPA-S-Si-Eu-phen@SiO_2_

The FT-IR spectra of SiO_2_, phen, SiO_2_@AIPA-S-Si-Eu-phen and SiO_2_@AIPA-S-Si-Eu-phen@SiO_2_ were shown in Fig. S5. In the FT-IR spectrum for phen (Fig. S5b), the stretching vibration of –C=N− appeared at 1589 cm^−1^, and the bending vibration of C-H appeared at 740 cm^−1^ and 856 cm^−1^ ^35,36^. In the FT-IR spectrum for SiO_2_@AIPA-S-Si-Eu-phen (Fig. S5c), the corresponding characteristic absorption bands are red shifted to 1566 cm^−1^(ν_C=N_), 725 and 848 cm^−1^(δ_C-H_), respectively. The above results demonstrate the success of the coordination of the Eu^3+^ ions with the nitrogen atoms in the second ligand phen. As can be observed in the FT-IR spectrum of SiO_2_@AIPA-S-Si-Eu-phen@SiO_2_ nanocomposite (Fig. S5d), the absorption band of –C=N− is significantly weakened and the characteristic absorption band of SiO_2_ appears in 1095 cm^−1^ (v_Si-O-Si_) and 950 cm^−1^ (v_Si-OH_), which further demonstrated that amorphous SiO_2_ is successfully coated on the surface of core-shell structured nanocomposite.

### XRD analysis

In order to study the structure of nanocomposites, the x-ray diffraction (XRD) patterns of all samples were tested from 5° to 80° at room temperature. As shown in Fig. S6, all samples exhibited similar wide diffraction peak at approximately 23° without any shift, except for a slight difference in intensity. The wide diffraction peak belongs to the amorphous SiO_2_^37,38^. The SiO_2_@AIPA-S-Si (Fig. S6 (A,B)b) was the same as the pure SiO_2_ (Fig. S6 (A,B)a) peak shape at about 23°, indicating that the first ligand (AIPA-S-Si) was grafted on the SiO_2_ surface without changing the structure of the SiO_2_. The peak shape of the core-shell structured nanocomposites SiO_2_@AIPA-S-Si-Eu (Fig. S6Ac) and SiO_2_@AIPA-S-Si-Eu-phen (Fig. S6Bc) were different from SiO_2_@AIPA-S-Si. It was noticed that a new diffraction peak appears at about 10°, which may be attributed to the formation of the Eu complex. Fig. S6Bc indicates that SiO_2_@AIPA-S-Si-Eu-phen nanocomposite shows some small diffraction peaks at 5–30°, while the diffraction peaks of amorphous SiO_2_ at about 23° were significantly weakened, further indicating that the rare earth complexes coated on the surface of SiO_2_ microspheres. Moreover, the peak shape of the core-shell-shell structured nanocomposites SiO_2_@AIPA-S-Si-Eu@SiO_2_ (Fig. S6Ad) and SiO_2_@AIPA-S-Si-Eu-phen@SiO_2_ (Fig. S6Bd) are similar to the pure SiO_2_ peak shape, indicating that amorphous SiO_2_ was successfully coated on the surface of the core-shell structured nanocomposite.

### TG analysis

In order to investigate core-shell and core-shell-shell structured nanocomposites have good thermal stability. The thermogravimetric analysis (TGA) curves of the core-shell and core-shell-shell structured nanocomposites were performed from 30 °C to 950 °C at a rate of 20 °C/min under N_2_ atmosphere. The thermogravimetric curve of core-shell structured SiO_2_@AIPA-S-Si-Eu-phen (a) and core-shell-shell structured SiO_2_@AIPA-S-Si-Eu-phen@SiO_2_ (b) were shown in Fig. S7. It can be observed that two nanocomposites have similar trends in weight loss and there were two major degradation steps. The first stage exhibited a weight loss of 3% and 6% for core-shell and core-shell-shell structured nanocomposites, respectively, which were attributed to the loss of water molecules physically adsorbed on the surface of the nanocomposites^39^. In the second stage, the weight loss of core-shell and core-shell-shell structured nanocomposites were 28% and 12%, respectively, which were due to the decomposition of organic complex. In general, the total weight loss percentage of core-shell and core-shell-shell structured nanocomposites were 31% and 18% from 34 °C to 670 °C. It was indicated that the percentage of organic complex in the core-shell structured nanocomposite is higher than that of the core-shell-shell structured nanocomposite, which further proves that amorphous SiO_2_ was coated on the surface of the core-shell structured nanocomposite. In addition, the second stage organic complex of the core-shell and core-shell-shell structured nanocomposites began to decompose at temperatures of 125 °C and 161 °C, respectively. The result indicates that the decomposition temperature of the core-shell-shell structured nanocomposite is higher than the core-shell structured nanocomposite. Therefore, the thermal stability of the core-shell-shell structured nanocomposite is higher than that of the core-shell structured nanocomposite. At the same time, because the core-shell-shell structured nanocomposite has the protection of SiO_2_ shell, it has a good promotion effect on thermal stability of nanocomposite.

### XPS analysis

**Figure 4:**
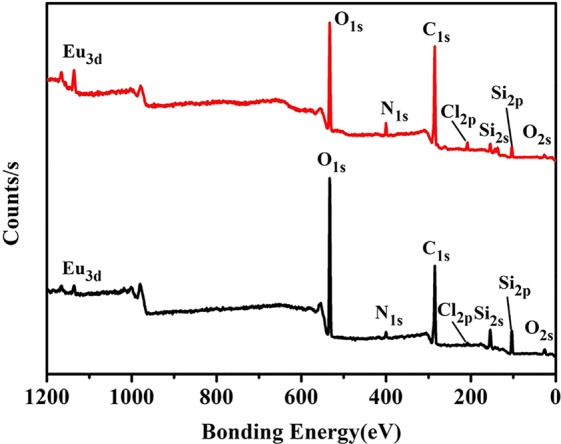
The XPS survey spectra of SiO_2_@AIPA-S-Si-Eu (**a**) and SiO_2_@AIPA-S-Si-Eu-phen (**b**).

The surface chemical compositions of the core-shell structured nanocomposites SiO_2_@AIPA-S-Si-Eu and SiO_2_@AIPA-S-Si-Eu-phen were further confirmed by XPS testing. The survey scan ranging from 0 to 1200 eV was shown in Fig. 4, the results demonstrated that core-shell structured nanocomposites were mainly composed by the elements, including C, N, O,Si, Cl and Eu. As shown in the XPS spectra (Fig. S8) of core-shell structured nanocomposites, the binding energy of C (1 s, 284.78 eV), (1 s, 288.65 eV); N (1 s, 399.3 eV); O (1 s, 532.45 eV); Si (2p, 103.35 eV); Cl (2p_1/2_, 209.25 eV), (2p_3/2_, 207.65 eV) and Eu (3d_5/2_, 1135.65 eV), (3d_3/2_, 1165.75 eV) can be clearly seen, respectively^40–43^. However, since XPS is a technique for surface detection and its detection depth is less than 10 nm, the presence of an element of N, O, Si, Cl and Eu indicates that the Eu complex was coated on the surface of the silica microspheres^44^. The above data further indicates that the core-shell structured nanocomposites SiO_2_@AIPA-S-Si-Eu and SiO_2_@AIPA-S-Si-Eu-phen have been successfully synthesized.

### Solid state luminescence properties

**Figure 5:**
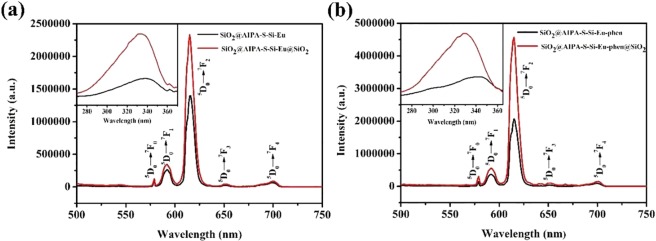
Emission spectra of (**a**) SiO_2_@AIPA-S-Si-Eu (black) and SiO_2_@AIPA-S-Si-Eu@SiO_2_ (red), (**b**) SiO_2_@AIPA-S-Si-Eu-phen (black) and SiO_2_@AIPA-S-Si-Eu-phen@SiO_2_ (red). Inset shows the corresponding Excitation spectra.

The excitation and emission spectra of the core-shell and core-shell-shell structured nanocomposites were measured in solid state by FLS-980 luminescence spectrometer at room temperature. The slit width of the nanocomposites SiO_2_@AIPA-S-Si-Eu, SiO_2_@AIPA-S-Si-Eu-phen, SiO_2_@AIPA-S-Si-Eu@SiO_2_ and SiO_2_@AIPA-S-Si-Eu-phen@SiO_2_ were 1, 0.7, 1, and 0.7 nm respectively. The luminescence intensity comparison of the core-shell and core-shell-shell structured nanocomposites were shown in Fig. 5. All nanocomposites exhibit similar luminescence spectra except for changes in intensity. The excitation spectra of all nanocomposites were recorded by monitoring at emission wavelength of 615 nm. The spectra were dominated by a broad excitation band centered at about 270–370 nm in ultraviolet region. It was designated as ligands that are absorbed into their π→π* transitions^37^. As shown in Fig. 5, five characteristic emission peaks were observed at 579 nm, 591 nm, 615 nm, 652 nm and 701 nm which represented ^5^D_0_→^7^F_J_ (J = 0, 1, 2, 3, 4) transitions of Eu^3+^ ions within 4f ^6^ configuration, respectively. The strongest emission peak at 615 nm was attributed to the ^5^D_0_→^7^F_2_ electrical dipole transition of Eu^3+^ ion, which was caused by the lack of inversion symmetry at Eu^3+^ ions site^45,46^. So, the strong red luminescence can be observed in the emisssion spectra. It can be seen from Fig. 5a that the luminescence intensities of core-shell structured nanocomposite SiO_2_@AIPA-S-Si-Eu was 1397610 a.u. Figure 5b shows that the luminescence intensity of the core-shell structured nanocomposite SiO_2_@AIPA-S-Si-Eu-phen was 2072052 a.u., which was 1.48 times higher than that of the core-shell structured nanocomposite SiO_2_@AIPA-S-Si-Eu. This is due to the addition of the second ligand (phen) can coordinate with the Eu^3+^ ion and improve the energy transfer efficiency, so that the nanocomposite luminescence intensity was significantly improved. On the other hand, the luminescence intensity of the core-shell-shell structured nanocomposite was stronger than that of the corresponding core-shell structured nanocomposite. From the data in Table 1, it was indicated that the two core-shell-shell structured nanocomposites increase by 1.67 times and 2.21 times compared with the corresponding core-shell structured nanocomposites, respectively. Since the core-shell structured nanocomposites were coated with a silica shell, which the high vibration energies loss of the ligand molecules (AIPA-S-Si, phen) or other quenching sites located at the surface of the core-shell structured nanocomposite could largely reduce. The silica shell can protect the rare earth complexes from quenching by the external environment^21^. Thus, the luminous intensity of the core-shell-shell structured nanocomposite is stronger than that of the corresponding core-shell structured nanocomposite. Luminescence intensity comparison data for all nanocomposites were shown in Table 1.

**Table 1:** Luminescence emission spectra data of core-shell and core-shell-shell nanocomposites.

nanocomposite	Slit width (nm)	λ_Ex_ (nm)	λ_Em_ (nm)	I (a. u.)	Intensity Changes
SiO_2_@AIPA-S-Si-Eu	1	339	615	1,397,610	
SiO_2_@AIPA-S-Si-Eu@SiO_2_	1	322	615	2,331,890	1.67
SiO_2_@AIPA-S-Si-Eu-phen	0.7	340	615	2,072,052	
SiO_2_@AIPA-S-Si-Eu-phen@SiO_2_	0.7	328	615	4,571,858	2.21

### Aqueous solution luminescence properties

**Figure 6:**
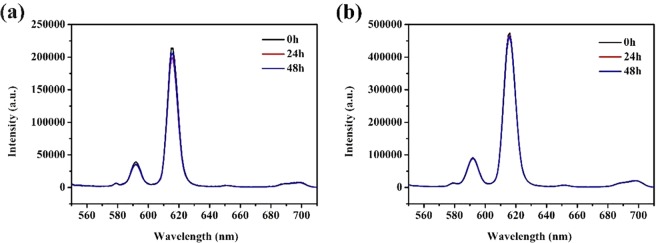
Emission spectra of (**a**) SiO_2_@AIPA-S-Si-Eu@SiO_2_ and (**b**) SiO_2_@AIPA-S-Si-Eu-phen@SiO_2_ after placement for 0 h, 24 h, 48 h in aqueous solution.

In order to further research the potential application of core-shell-shell structured nanocomposites in the biological field, we have tested the stability of core-shell-shell structured nanocomposites in aqueous solution. The core-shell-shell structured nanocomposites were dissolved in distilled water to prepare a suspension having a concentration of 0.1 g/L. The prepared solution was placed for 0 h, 24 h, 48 h and the luminescence intensity was measured. Figure 6 shows that the luminescence intensity of the nanocomposites in the aqueous still has strong luminescence intensity and no significant change after being placed for different time. Therefore, the core-shell-shell structured nanocomposites have excellent luminescence stability in aqueous solution.

Fig. S9 shows the calculated CIE chromaticity coordinates of all nanocomposites investigated. SiO_2_@AIPA-S-Si-Eu, SiO_2_@AIPA-S-Si-Eu@SiO_2_, SiO_2_@AIPA-S-Si-Eu-phen and SiO_2_@AIPA-S-Si-Eu-phen@SiO_2_ show a red emission located at (0.5152, 0.2655), (0.4893, 0.2536), (0.5736, 0.3124) and (0.5917, 0.3212) respectively. The results of chromaticity coordinates show that all the nanocomposites are located in the red or pink region.

**Figure 7:**
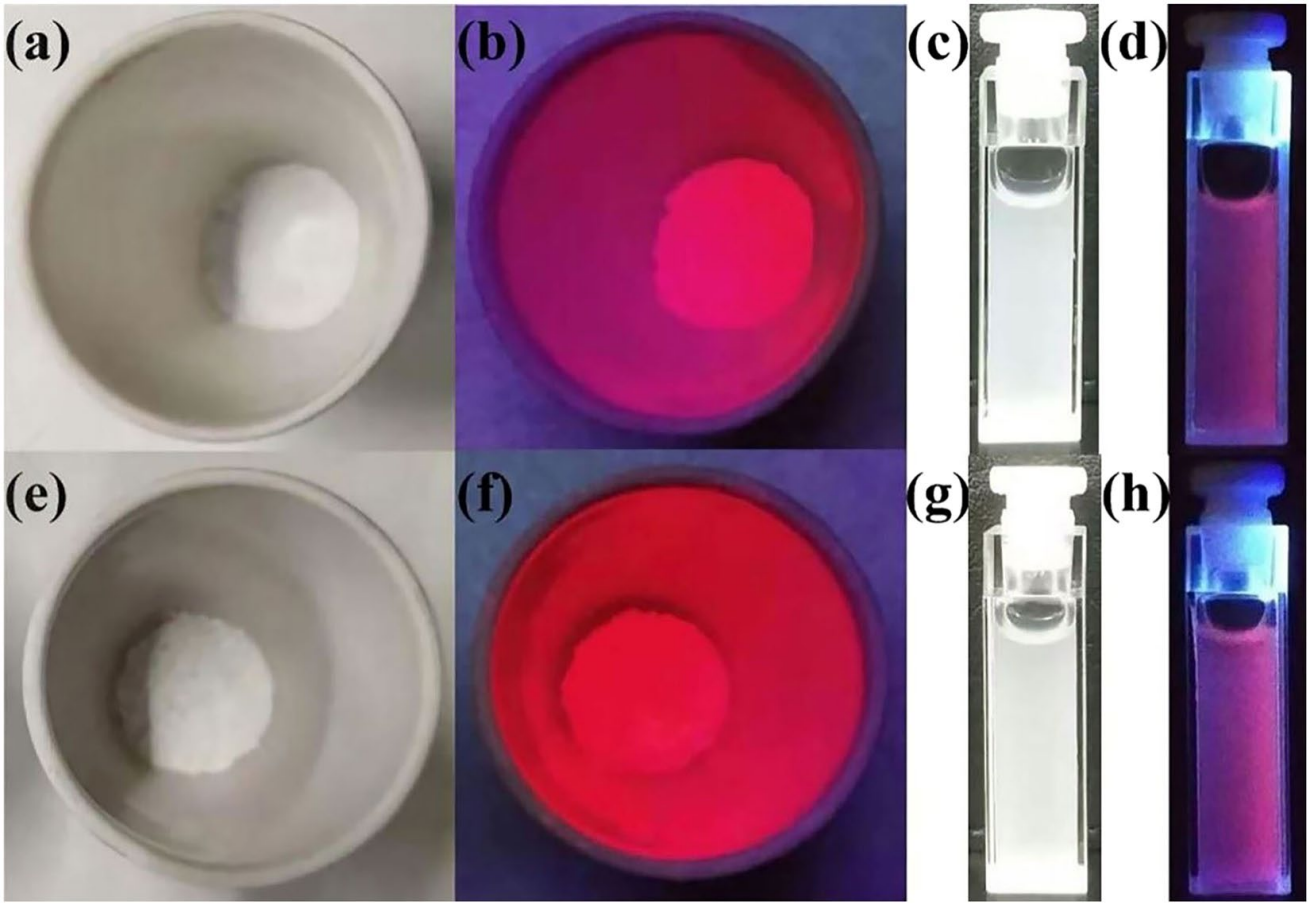
The images of SiO_2_@AIPA-S-Si-Eu@SiO_2_ (**a,b**) and SiO_2_@AIPA-S-Si-Eu-phen@SiO_2_ (**e,f**) without (**a,e**) and under UV irradiation (**b,f**). The images of SiO_2_@AIPA-S-Si-Eu@SiO_2_ (**c,d**) and SiO_2_@AIPA-S-Si-Eu-phen@SiO_2_ (**g,h**) dispersed in water without (**c,g**) and under UV irradiation (**d,h**).

Figure 7 exhibits, as an example, the red light image of SiO_2_@AIPA-S-Si-Eu@SiO_2_ (a,b) and SiO_2_@AIPA-S-Si-Eu-phen@SiO_2_ (e,f) nanocomposites without and under UV irradiation. In addition, the image also show the pink light image of a solution of SiO_2_@AIPA-S-Si-Eu@SiO_2_ (c,d) and SiO_2_@AIPA-S-Si-Eu-phen@SiO_2_ (g,h) nanocomposites dispersed in water without and under UV irradiation. Under ultraviolet irradiation, core-shell-shell structured nanocomposites exhibit bright red or pink light in solid or aqueous solution, which indicates that core-shell-shell structured nanocomposites have excellent luminous properties.

### The luminescence lifetime

To further study the luminescence properties, the lifetime curve of nanocomposites was also measured at room temperature. As shown in Fig. S10, all curves follow a double exponential fit (1)^38^:

1 $${{\rm{I}}}_{({\rm{t}})}={{\rm{I}}}_{0}+{{\rm{A}}}_{1}\,\exp \,(-{\rm{t}}/{{\rm{t}}}_{1})+{{\rm{A}}}_{2}\,\exp \,(-{\rm{t}}/{{\rm{t}}}_{2})$$

where I_(t)_ and I_0_ are the luminescence intensities, A_1_ and A_2_ are the fitting parameters, and t_1_ and t_2_ are the decay time, respectively. In addcay lifetime (t) is calculated using Eq. (2):

2 $${\rm{t}}=({{\rm{A}}}_{1}{{{\rm{t}}}_{1}}^{2}+{{\rm{A}}}_{2}{{{\rm{t}}}_{2}}^{2})/({{\rm{A}}}_{1}{{\rm{t}}}_{1}+{{\rm{A}}}_{2}{{\rm{t}}}_{2})$$

Thus, the average liftime were detetmined to be 0.63, 1.10, 0.64, and 0.58 ms for the SiO_2_@AIPA-S-Si-Eu, SiO_2_@AIPA-S-Si-Eu@SiO_2_, SiO_2_@AIPA-S-Si-Eu-phen and SiO_2_@AIPA-S-Si-Eu-phen@SiO_2_. It can be found that all nanocomposites have a long lifetime.

### Low temperature phosphorescence spectra

To further explain the luminescence mechanism of the nanocomposites. The low phosphorescence spectra of the ligands were tested on a FLS-980 phosphorescence spectrometer at 77 K. The excitation wavelengths of the ligands AIPA-S-Si and phen were 355 nm and 372 nm, respectively. The corresponding slit width was 1 nm and 10 nm, respectively. The triplet energy level of the ligand was determined by the half width of the band. In the phosphorescence spectra of the first ligand AIPA-S-Si (Fig. S11a), there was an emission band of 455 nm to 530 nm, indicating that the triplet energy level was 21978 cm^−1^ to 18868 cm^−1^. The excited state energy level ^5^D_0_ of Eu^3+^ ion was calculated to be 17277 cm^−1^. Therefore, the triplet energy level of the first ligand AIPA-S-Si was higher than the excitable energy level of the Eu^3+^ ion, and the ligand can effectively transfer the energy absorbed from the ultraviolet region to the central Eu^3+^ ions, thereby sensitizing the luminescence of the Eu^3+^ ion. In the phosphorescence spectrum of the second ligand phen, the emission band from 503 nm to 618 nm can be observed from the Fig. S11b. The corresponding triplet energy level was 19881 cm^−1^ to 16181 cm^−1^. The highest triplet energy level of phen was also higher than the excitable energy level of Eu^3+^ ions, so the luminescence of Eu^3+^ ions can also be sensitized. The schematic diagram of the triplet energy levels and Eu^3+^ ions excited state levels of the ligands AIPA-S-Si and phen was shown in Fig. S12. Since the first ligand AIPA-S-Si and the second ligand phen can synergistically sensitize the Eu^3+^ ions. Therefore, the luminescence intensity of the SiO_2_@AIPA-S-Si-Eu-phen nanocomposite was superior to that of the SiO_2_@AIPA-S-Si-Eu nanocomposite. In summary, the presence of the ligands AIPA-S-Si and phen can sensitize Eu^3+^ ions to have excellent luminescent properties.

## Conclusions

The four novel nanocomposites SiO_2_@AIPA-S-Si-Eu, SiO_2_@AIPA-S-Si-Eu-phen, SiO_2_@AIPA-S-Si-Eu@SiO_2_ and SiO_2_@AIPA-S-Si-Eu-phen@SiO_2_ have been successfully prepared; All nanocomposites have excellent luminescence properties, and the core-shell-shell structured nanocomposites have stronger luminescence intensity than the corresponding core-shell structured nanocomposites; The aqueous solution of core-shell-shell structured nanocomposites have excellent luminescence stability, and the luminescence intensity is almost unchanged after being placed for 0 h, 24 h and 48 h at room temperature; Besides, the core-shell and core-shell-shell structured nanocomposites can also save rare earth resources and reduce production cost in the actual production process; More importantly, the silanol groups on the surface of the core-shell-shell structured nanocomposites are easily attached to biomolecule. Consequently, the research of core-shell-shell structured nanocomposites not only has potential applications in biology, but also excellent luminescent properties provide new research ideas for other luminescent materials.

## Materials and Methods

### Materials

5-amino-iso-phthalic acid (AIPA, 99.8%, Aldrich), cetyltrimethylammonium bromide (CTAB), acetone (C_3_H_4_O, 99%), 3-(triethoxysilyl)-propyl isocyanate (TEPIC, 96%, Aldrich), Europium oxide (Eu_2_O_3_, 99.999%), perchloric acid (HClO_4_, 1 mol/L), Europium perchlorate (Eu(ClO_4_)_3_.nH_2_O) was prepared by Eu_2_O_3_ and HClO_4_. Tetraethyl orthosilicate (TEOS, 99%), ammonia solution (NH_3_.H_2_O, 25%), 1,10-phenanthroline (phen, 99%), absolute ethanol (C_2_H_5_OH, 99%). All chemical reagents are of analytical grade.

### Synthesis of ligand (AIPA-S-Si)

First, 2 mmol 5-aminoisophthalic acid (AIPA) was placed in a 100 mL dry three-neck flask, and 40 mL acetone was added to completely dissolve it. Then, 6 mmol 3-(triethoxysilyl)-propyl isocyanate (TEPIC) was added to the above solution, and reflux reaction was continued at 55 °C for 15 h to ensure complete reaction. It was clearly observed that a large amount of white precipitate was produced at the bottom of the flask. Subsequently, the remaining solvent was evaporated to dryness, washed three times with cold n-hexane. Finally, the product was dried at 60 °C for 5 h to give a white powder. The product is defined as AIPA-S-Si (C_18_H_28_N_2_O_8_Si, M = 428 g/mol). Yield: 80%. The elemental analysis results are as follows: C 50.36%, H 6.35%, N 6.61%; Found: C 50.47%, H 6.54%, N 6.54%. The ^1^H NMR (DMSO as a solvent) data are as follows (Fig. S13): δ 13.13 (s, 2H, –COOH), δ 8.88 (s, 1H, –NH–), δ 8.22 (s, 1H, –CH–), δ 8.01 (s, 1H, CH–), δ 6.27 (t, 1H, –NH–), δ 3.76 (q, 6H, –CH_2_–), δ 3.06 (q, 2H, –CH_2_−), δ 1.5 (m, 2H, –CH_2_–), δ 1.14 (t, 9H, –CH_3_), δ 0.56 (t, 2H, –CH_2_–).

### Synthesis of SiO_2_ microspheres

The SiO_2_ microspheres were prepared according to the Stöber method^47^. Specifically, 1.7 mL ammonia water and 4 mL distilled water were added to 35 mL absolute ethanol, and then 1.7 mL TEOS was added dropwise to the above solution under magnetic stirring. Then, a white suspension was obtained after continuing the reaction for 6 h at room temperature. The resulting product was centrifuged, washed with ethanol and distilled water several times, respectively. Finally, SiO_2_ microspheres were dried at 50 °C for 5 h.

### Synthesis of SiO_2_@AIPA-S-Si

Briefly, 0.1 g as-prepared SiO_2_ microspheres were accurately weighed and dissolved in a mixed solution of 10 mL absolute ethanol and 10 mL distilled water, and then the as-prepared ligand (AIPA-S-Si) was ultrasonically dissolved in 20 mL 1,4-dioxane. Subsequently, the ligand (AIPA-S-Si) solution was gradually added dropwise to the silica solution, and the reaction was stirred at room temperature for 36 h. The product was washed three times with absolute ethanol and collected by centrifugation at 8000 r. min^−1^. Finally, the product was dried at 50 °C for 10 h. The product is defined as SiO_2_@AIPA-S-Si.

### Synthesis of SiO_2_@AIPA-S-Si-Eu and SiO_2_@AIPA-S-Si-Eu@SiO_2_

Typically, 0.0550 g as-prepared SiO_2_@AIPA-S-Si was uniformly dispersed in 15 mL absolute ethanol. Subsequently, 0.0550 g Eu(ClO_4_)_3_.nH_2_O was dissolved in 5 mL absolute ethanol, which was added dropwise to the above solution, and fully reacted under magnetic stirring at 50 °C for 24 h. The obtained product was centrifuged, washed with absolute ethanol several times, and the white product was dried at 50 °C for 5 h. The product is defined as SiO_2_@AIPA-S-Si-Eu.

0.02 g SiO_2_@AIPA-S-Si-Eu and 0.01 g CTAB were dissolved in 20 mL of a mixed solvent (5 mL distilled water, 15 mL ethanol), then add the appropriate amount of ammonia water and 15 μL TEOS. Then, the mixture was continuously stirred at room temperature for 24 h. The resulting suspension was washed six times with ethanol and water. After centrifugation at 8000 r.min^−1^, the final product was dried at 50 °C for 5 h. The product is defined as SiO_2_@AIPA-S-Si-Eu@SiO_2_.

### Synthesis of SiO_2_@AIPA-S-Si-Eu-phen and SiO_2_@AIPA-S-Si-Eu-phen@SiO_2_

The synthesis method was identical to the above method. 0.05 g SiO_2_@AIPA-S-Si-Eu and 0.04 g phen were ultrasonically dispersed in 15 mL absolute ethanol, 0.7 g Eu(ClO_4_)_3_.nH_2_O was dissolved in 5 mL absolute ethanol, and then slowly dropped into the above solution. The reaction was carried out under magnetic stirring at 50 °C for 24 h. The white powder obtained after centrifugation, washing and drying was defined as SiO_2_@AIPA-S-Si-Eu-phen. Correspondingly, the core-shell-shell structured nanocomposite was synthesized in the same method as above, and the obtained product is defined as SiO_2_@AIPA-S-Si-Eu-phen@SiO_2_.

### Characterization

^1^H nuclear magnetic resonance (NMR) spectrum was recorded on a Bruker Advancell spectrometer with DMSO as the solvent. All samples were analyzed by FT-IR spectroscopy, which were measured by a Bruker VERTEX70 FT-IR spectrophotometer in the wave number range of 400–4000 cm^−1^ using potassium bromide (KBr) pellets. The X-ray diffraction (XRD) analysis was recorded using a PANalytical Empyrean diffractometer, which equipped with Cu Kalpha1 radiation (1.5405 Å), the scanning rate was 15°/min in the 2θ range of 5–80° at room temperature. The morphology of the nanocomposites was characterized on Hitachi S-4800 scanning electron microscopy (SEM) at 20 kV and FEI Tecnai F20 transmission electron microscopy (TEM) at an acceleration voltage of 200 kV. Thermogravimetric analysis (TGA) was tested on a STA6000 thermogravimetric analyzer in air at a heating rate of 20 °C/min from room temperature to 950 °C. Luminescence spectra were tested at room temperature using a FLS-980 (Edinburgh Instruments, England) with a xenon lamp as the light source. Phosphorescence spectra were measured at 355 nm and 372 nm excitation on a FLS-980 spectrofluorometer at 77 K. X-ray photoelectron spectroscopy (XPS) spectra were recorded on Thermo ESCALAB 250Xi spectrometer (Thermo Fisher Scientific, Waltham, MA, USA).

## Supplementary information

Supplementary information.
