# Supplementary material for: Controlled synthesis and luminescence properties of core-shell-shell structured SiO2@AIPA-S-Si-Eu@SiO2 and SiO2@AIPA-S-Si-Eu-phen@SiO2 nanocomposites
Source: Sci Rep. 2020 Feb 26;10:3522. doi: 10.1038/s41598-020-60538-w (PMC7044297; doi:10.1038/s41598-020-60538-w)
Supplement: Supplementary file 1 — Supplementary information. [file 41598_2020_60538_MOESM1_ESM.pdf]

Supporting Information

Controlled synthesis and luminescence properties of core-shell-shell structrued SiO_2@AIPA-S-Si-Eu@SiO_2 and SiO_2@AIPA-S-Si-Eu-phen@SiO_2 nanocomposites

Yan Qiao [1], Wenxian Li [1,*], Jinrong Bao [1], Yushan Zheng [2], Lina Feng [1] ,Yangyang Ma [1], Kuisuo Yang [1], Anping Wu [1], He Bai [1], Yunjiang Yang [1]

[1] Inner Mongolia Key Laboratory of Chemistry and Physics of Rare Earth Materials, School of Chemistry and Chemical Engineering, Inner Mongolia University, Hohhot 010021, China

[2] Inner Mongolia Autonomous Region Food Inspection Test center, Hohhot 010021, China

[*] To whom the correspondence should be addressed. Tel: (+86)-0471-4990061. E-Mail: nmglwx@163.com (W. X. Li).

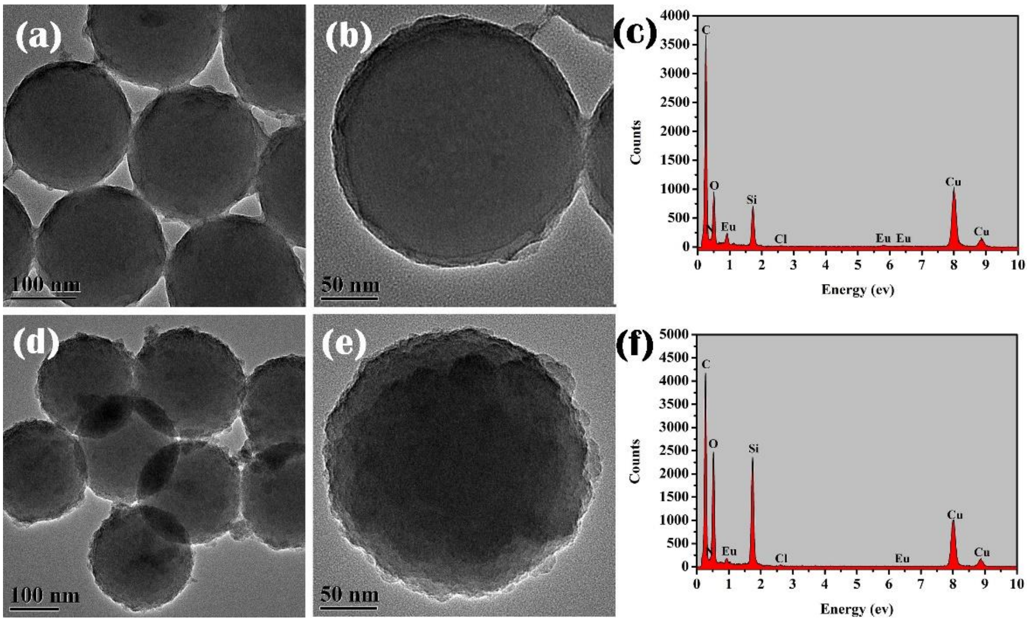

Figure S1. TEM images of SiO_2@AIPA-S-Si-Eu-phen (**a**,**b**) and SiO_2@AIPA-S-Si-Eu-phen@SiO_2 (**d**,**e**). EDX images of SiO_2@AIPA-S-Si-Eu-phen (**c**) and SiO_2@AIPA-S-Si-Eu-phen@SiO_2 (**f**).

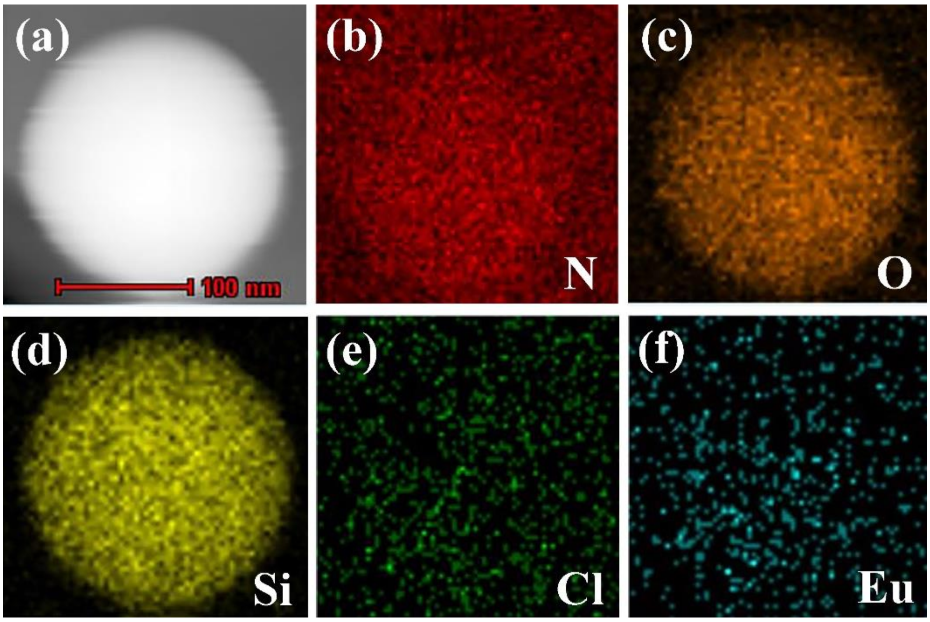

Figure S2. (**a**) HRTEM image of SiO_2@AIPA-S-Si-Eu-phen@SiO_2 nanocomposite and corresponding elemental mapping images represent the (**b**) nitrogen mapping, (**c**) theoxygen mapping, (**d**) silicon mapping, (**e**) chlorinum mapping, and (**f**) europium mapping.

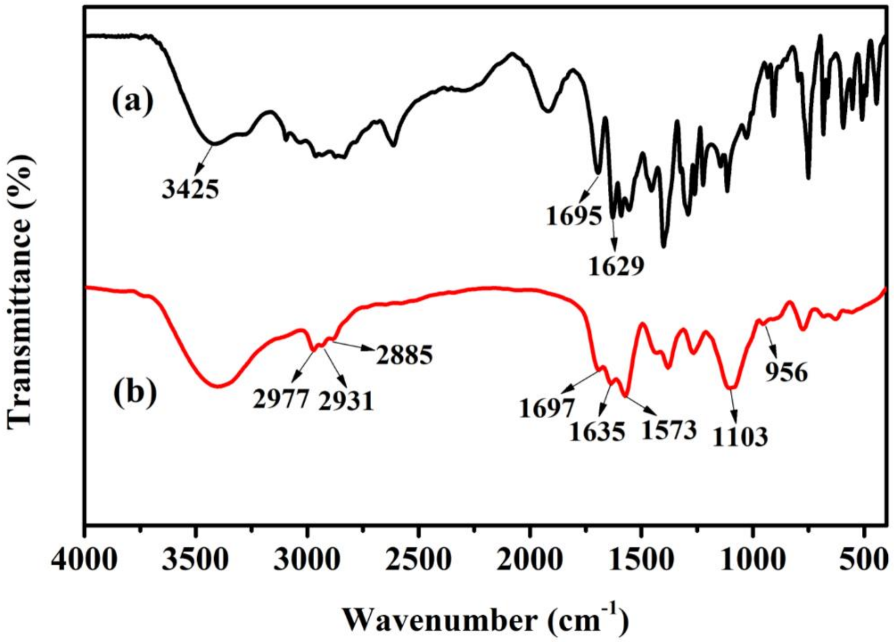

Figure S3. The FT-IR spectra of AIPA (**a**), AIPA-S-Si (**b**).

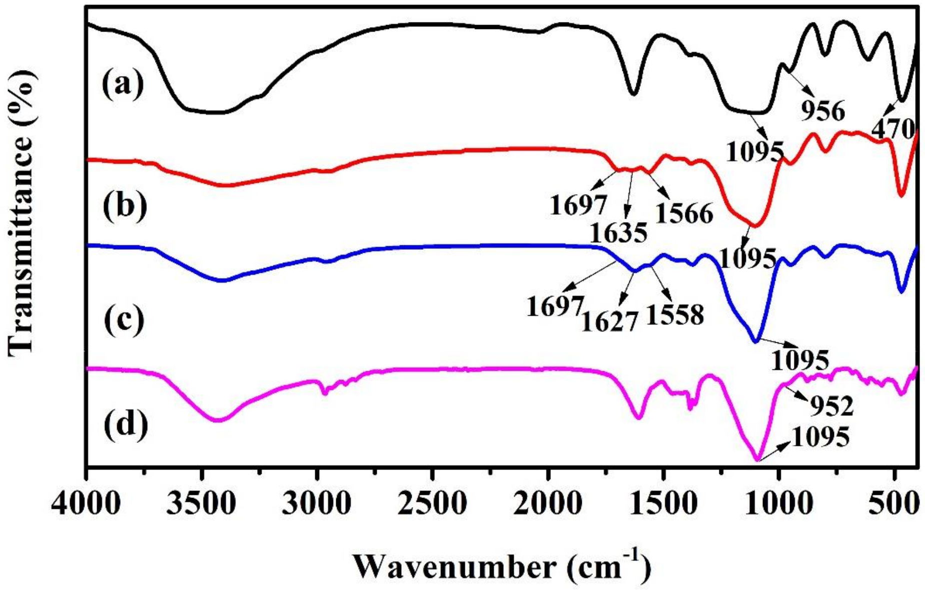

Figure S4. The FT-IR spectra of SiO_2 (**a**), SiO_2@AIPA-S-Si (**b**), SiO_2@AIPA-S-Si-Eu (**c**), SiO_2@AIPA-S-Si-Eu@SiO_2 (**d**).

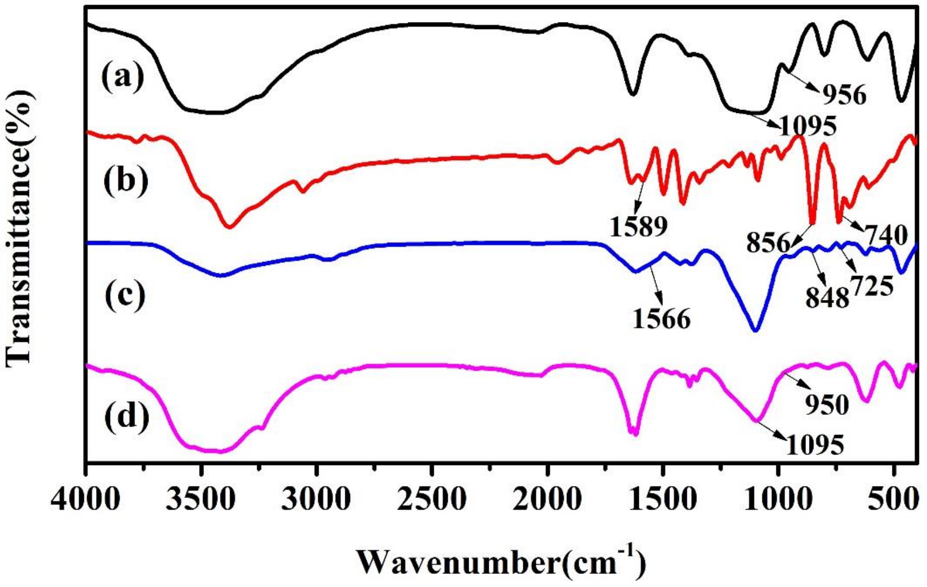

Figure S5. The FT-IR spectra of SiO_2 (**a**), phen (**b**), SiO_2@AIPA-S-Si-Eu-phen (**c**), SiO_2@AIPA-S-Si-Eu-phen@SiO_2 (**d**).

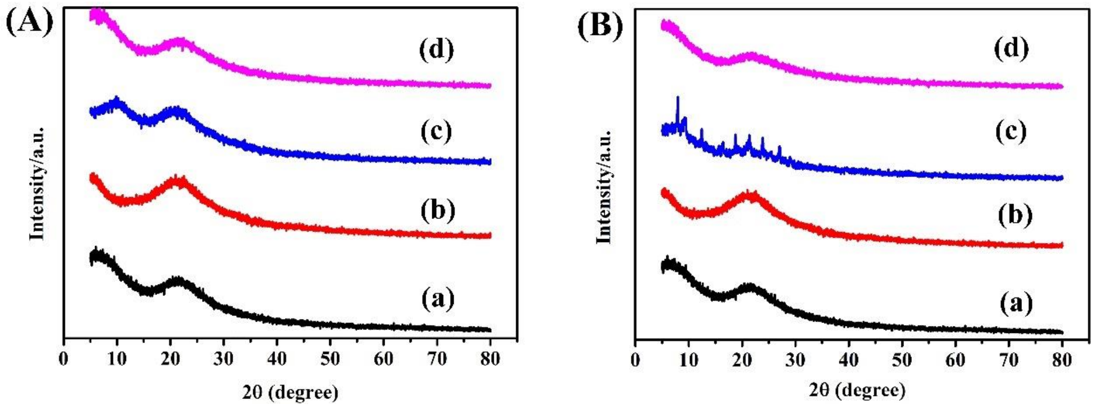

Figure S6. (**A**) XRD spectra of SiO_2 (**a**), SiO_2@AIPA-S-Si (**b**), SiO_2@AIPA-S-Si-Eu (**c**) and SiO_2@AIPA-S-Si-Eu@SiO_2 (**d**). (**B**) XRD spectra of SiO_2 (**a**), SiO_2@AIPA-S-Si (**b**), SiO_2@AIPA-S-Si-Eu-phen (**c**) and SiO_2@AIPA-S-Si-Eu-phen@SiO_2 (**d**).

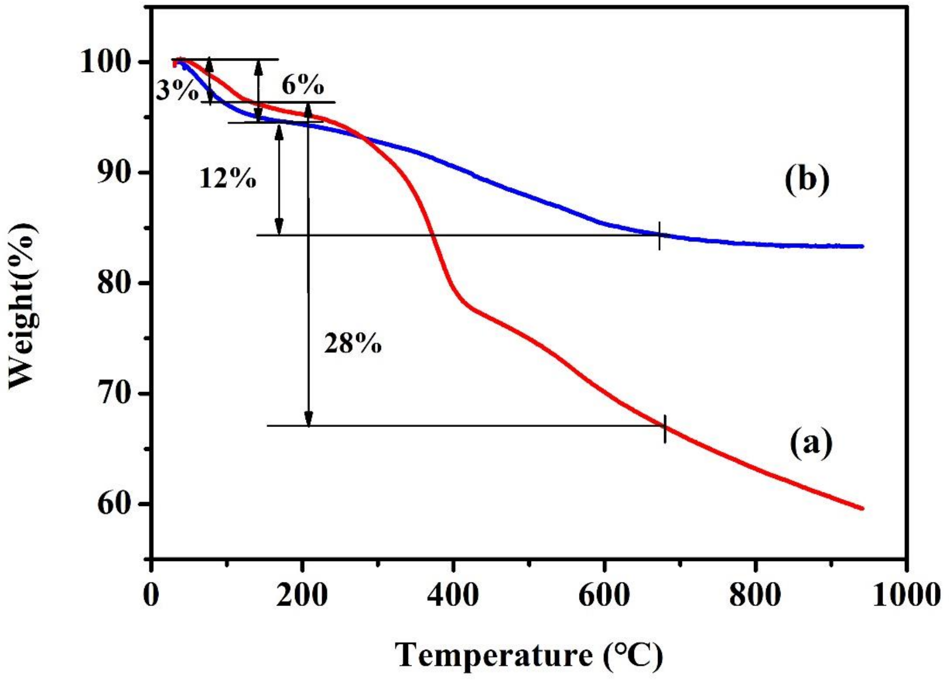

Figure S7. TGA curves of SiO_2@AIPA-S-Si-Eu-phen (**a**), SiO_2@AIPA-S-Si-Eu-phen@SiO_2 (**b**).

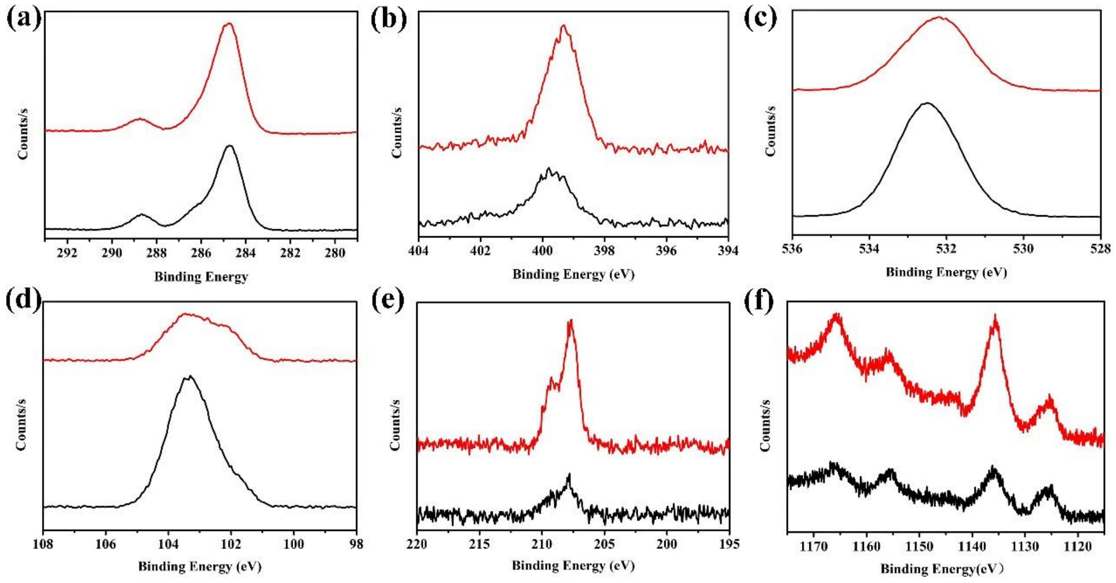

Figure S8. The high resolutoin spectra (**a**) C 1s region, (**b**) N 1s region, (**c**) O 1s region, (**d**) Si 2p region, (**e**) Cl 2p region, (**f**) Eu 3d region of SiO_2@AIPA-Si-Eu (black) and SiO_2@AIPA-Si-Eu-phen (red).

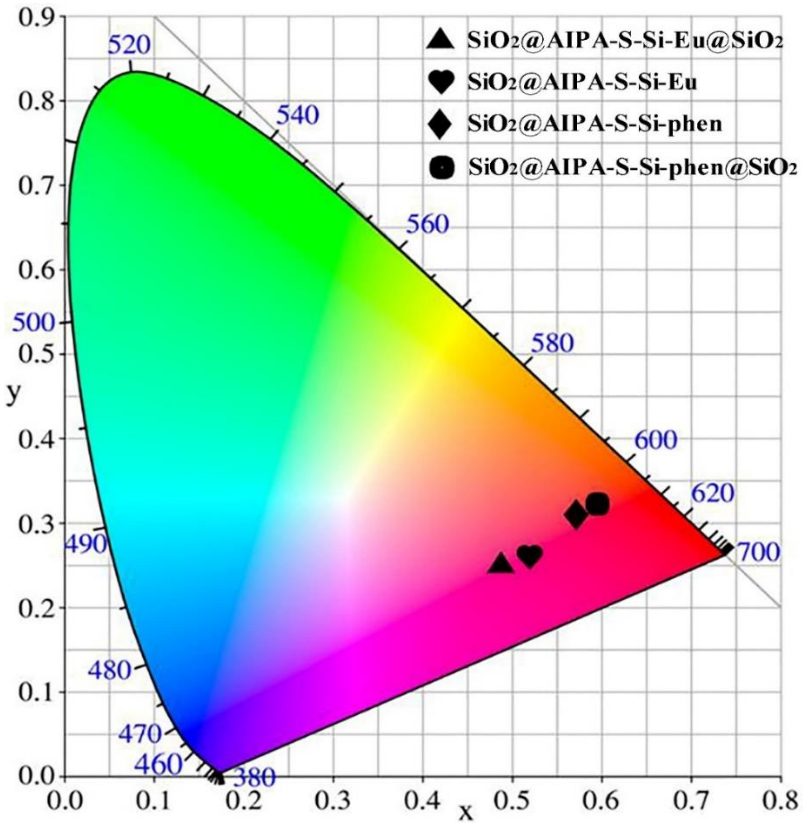

Figure S9. CIE chromaticity coordinates diagram of SiO_2@AIPA-S-Si-Eu, SiO_2@AIPA-S-Si-Eu@SiO_2, SiO_2@AIPA-S-Si-Eu-phen and SiO_2@AIPA-S-Si-Eu-phen@SiO_2.

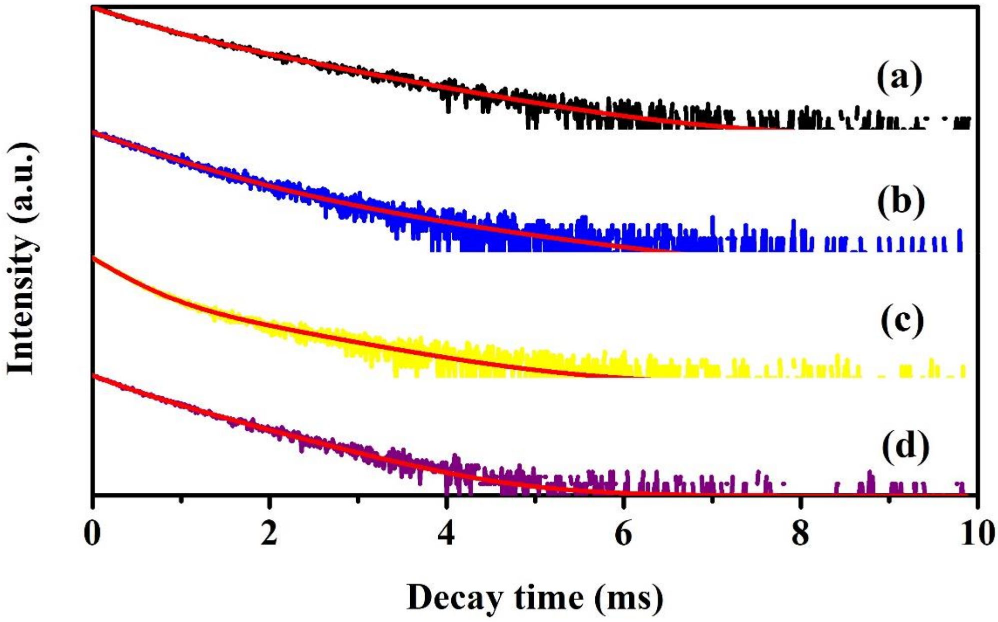

Figure S10. Decay curve of SiO_2@AIPA-S-Si-Eu (**a**), SiO_2@AIPA-S-Si-Eu@SiO_2 (**b**), SiO_2@AIPA-S-Si-Eu-phen (**c**) and SiO_2@AIPA-S-Si-Eu-phen@SiO_2 (**d**).

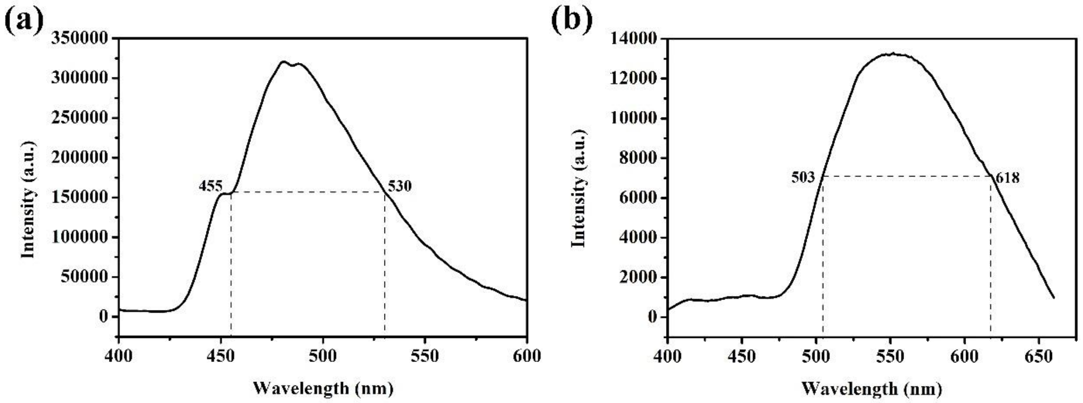

Figure S11. phosphorescence spectra of (**a**) AIPA-S-Si, (**b**) phen.

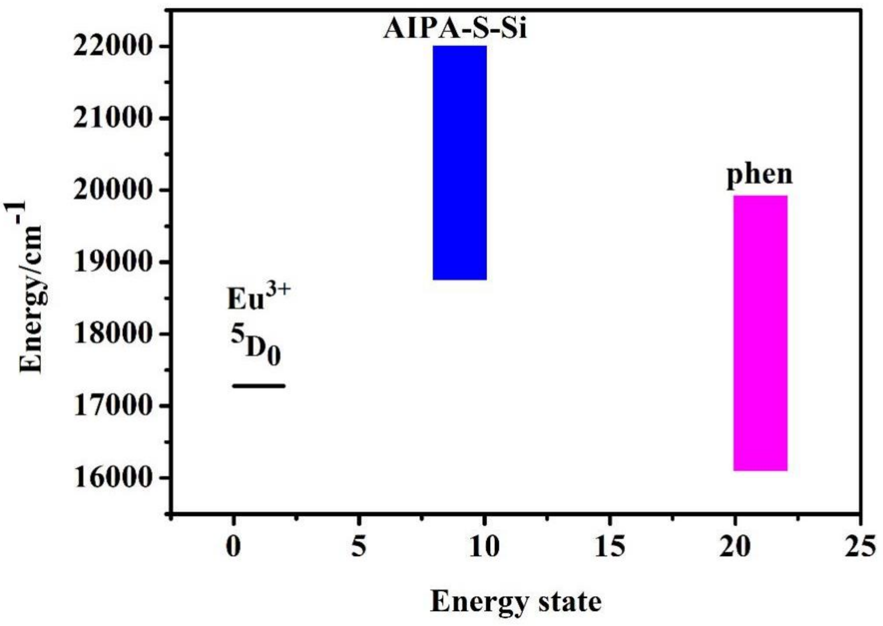

Figure S12. Triple state of ligands and the excited state of Eu^{3+}.

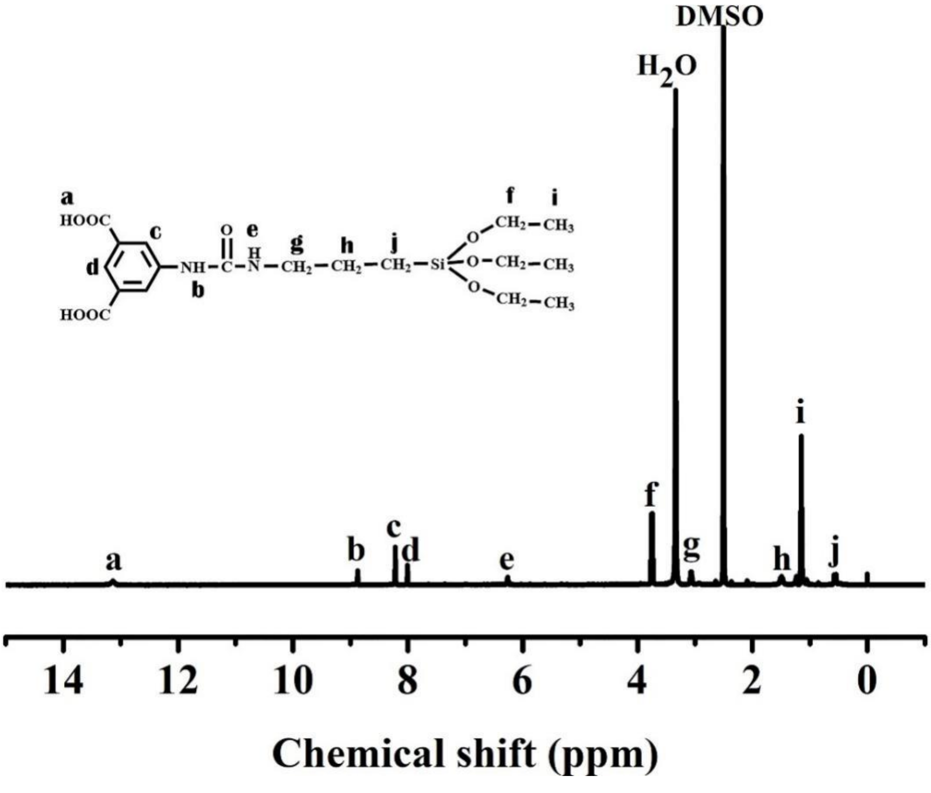

Figure S13. ^1H NMR spectra of AIPA-S-Si using DMSO as the solvent.
